# Transcript-Level Dysregulation of *BCL2* Family Genes in Acute Myeloblastic Leukemia

**DOI:** 10.3390/cancers13133175

**Published:** 2021-06-25

**Authors:** Luiza Handschuh, Pawel Wojciechowski, Maciej Kazmierczak, Krzysztof Lewandowski

**Affiliations:** 1 Laboratory of Genomics, Institute of Bioorganic Chemistry, Polish Academy of Sciences, 61-704 Poznan, Poland; 2 Institute of Computing Science, Poznan University of Technology, 60-965 Poznan, Poland; Pawel.Wojciechowski@cs.put.poznan.pl; 3 Department of Hematology and Bone Marrow Transplantation, Poznan University of Medical Sciences, 60-569 Poznan, Poland; maciej.kazmierczak@onet.eu (M.K.); krzysztof.lewandowski@skpp.edu.pl (K.L.)

**Keywords:** AML, *BCL2* family, apoptosis, gene expression, RNA-seq, exome sequencing, response to therapy, mutation, splicing, correlation

## Abstract

**Simple Summary:**

The BCL2 family comprises pro- and anti-apoptotic proteins whose cellular abundance, modifications, and interactions determine the cell fate. Its contribution to pathogenesis of acute myeloid leukemia (AML) was not widely studied and none of the studies published before presented a complex picture of *BCL2* family gene expression. Therefore, we decided to analyze the AML transcriptome sequencing data to outline this picture and look for relations between the expression of particular BCL2 family members and the genes encoding interacting proteins, presence of mutations, and clinical features. Our findings should not only shed light on apoptosis- and oncogenesis-related processes, but may also be implemented in clinical practice. Prognostic significance, association with response to therapy and potential application in the selection of therapeutic targets are of particular importance.

**Abstract:**

The expression of apoptosis-related *BCL2* family genes, fine-tuned in normal cells, is dysregulated in many neoplasms. In acute myeloid leukemia (AML), this problem has not been studied comprehensively. To address this issue, RNA-seq data were used to analyze the expression of 26 BCL2 family members in 27 AML FAB M1 and M2 patients, divided into subgroups differently responding to chemotherapy. A correlation analysis, analysis of variance, and Kaplan-Meier analysis were applied to associate the expression of particular genes with other gene expression, clinical features, and the presence of mutations detected by exome sequencing. The expression of *BCL2* family genes was dysregulated in AML, as compared to healthy controls. An upregulation of anti-apoptotic and downregulation of pro-apoptotic genes was observed, though only a decrease in *BMF*, *BNIP1*, and *HRK* was statistically significant. In a group of patients resistant to chemotherapy, overexpression of *BCL2L1* was manifested. In agreement with the literature data, our results reveal that *BCL2L1* is one of the key players in apoptosis regulation in different types of tumors. An exome sequencing data analysis indicates that *BCL2* family genes are not mutated in AML, but their expression is correlated with the mutational status of other genes, including those recurrently mutated in AML and splicing-related. High levels of some *BCL2* family members, in particular *BIK* and *BCL2L13*, were associated with poor outcome.

## 1. Introduction

Acute myeloid leukemia (AML) is a heterogeneous hematological malignancy which develops as a result of hematopoietic differentiation impairment and excessive proliferation of myeloid progenitor cell clones [1,2]. Their accumulation in the bone marrow, peripheral blood, and organs leads to the manifestation of systemic symptoms and death within weeks to months, if the disease is left untreated [3]. Chromosomal rearrangements, small somatic mutations and dysregulation of the gene expression have been described as AML hallmarks [4,5,6,7]. Similarly to solid tumor cells, leukemic cells present enhanced survival rate and successfully evade apoptosis [8]. Apoptosis or programmed cell death is involved in a natural cell turnover, embryonic development, and the elimination of impaired or aging cells [9]. Therefore, dysregulation of this process usually has severe consequences [8,10].

Proteins belonging to the BCL2 (B-Cell lymphoma 2) family regulate all major types of cell death, including apoptosis, necrosis, and autophagy; although they are the best known for contribution to the intrinsic (mitochondrial) apoptotic pathway [11,12,13,14]. Their cellular content, post-translational modifications, and interactions determine cell fate, deciding between survival and death [14,15,16]. Up to date, 25 different BCL2 family members have been identified and most of them have two or more isoforms [15,17,18]. The family founder, the *BCL2* gene, is involved in t(14;18) chromosomal translocation in B-cell lymphomas and frequently overexpressed in cancer [12,19,20]. The pro-apoptotic BCL2 family members include two subgroups: a “multi-domain” containing up to 4 BCL2 homology (BH1-BH4) domains and a “BH3-only” [12,20,21]. The multi-domain subgroup includes the BCL2 homologous antagonist/killer (BCL2-like protein 4 alias apoptosis regulator BAK, encoded by the *BAK1* gene); the apoptosis regulator BAX (BCL2-like protein 4, encoded by *BAX* gene); and the BCL2-related ovarian killer (BCL2-like protein 9, encoded by the *BOK* gene). These three proteins trigger apoptosis by forming pores within the mitochondrial outer membrane. Mitochondrial outer membrane permeabilization (MOMP) is necessary to release pro-apoptogenic factors (e.g., cytochrome c) from the mitochondrial intermembrane space (IMS) into the cytosol, which activates a caspase cascade [14]. The pro-apoptotic BH3-only protein subgroup consists of inter alia BCL2-associated death promoter (BAD, encoded by the *BAD* gene); BH3-interacting domain death agonist (BID, encoded by the *BID* gene); BCL2-like protein 11 (BIM, encoded by the *BCL2L11* gene); p53 upregulated modulator of apoptosis (PUMA, encoded by the *BBC3* gene); BCL2 modifying factor (encoded by the *BMF* gene), activator of apoptosis harakiri (encoded by the *HRK* gene); and NADPH oxidase activator 1 (NOXA, alias Phorbol-12-myristate-13-acetate-induced protein 1, encoded by the *PMAIP1* gene) [12,13,14,15]. The BH3-only proteins interact with anti-apoptotic proteins forming homo- or heterodimers, and the type of dimer partners determine the cell fate [13,14]. The anti-apoptotic BCL2 family members include apoptosis regulator BCL2 (encoded by the *BCL2* gene); B-cell lymphoma-extra-large (BCL-XL, alias BCL2-like protein 1, encoded by the *BCL2L1* gene); apoptosis regulator BCL-W (BCL2-like protein 2, encoded by the *BCL2L2* gene); induced myeloid leukemia cell differentiation protein Mcl-1 (encoded by the *MCL1* gene); and BCL2-related protein A1 (alias BFL-1, encoded by the *BCL2A1* gene). These proteins sequester pro-apoptotic BH3-only proteins, thereby preventing the activation of multi-domain pro-apoptotic proteins [22,23].

The expression of genes encoding the BCL2 family proteins is frequently dysregulated in cancer [12]. The up-regulation of pro-survival *BCL2* genes has also been associated with the pathogenesis of AML [24,25,26,27]. Thus, BCL2-targeted therapy has recently been introduced to cancer treatment with promising results [28,29]. In the AML induction treatment, the administration of anthracycline and cytosine arabinoside (3 + 7 regimen) has been used for more than 40 years [30,31,32]. Approximately 40–45% of younger and only 10–20% of older adults with AML may be cured with the current standard chemotherapy [33]. Despite the progress in the understanding of the disease biology, the three-year overall survival (OS) of AML patients is unacceptably low (~10%) in the case of relapsed or refractory (R/R) disease [1,34]. The allogenic stem cell transplantation remains the sole curative option, but is not always available [33]. Seventy percent of patients aged >65 die within 1 year from the moment of diagnosis [2]. Therefore, a lot of efforts have been made to individualize AML therapy according to the molecular profile of AML blast cells [35,36]. The application of next generation sequencing (NGS) has opened up new possibilities to precisely diagnose the disease, look for molecular bases of AML refractoriness, and try to overcome it. The introduction of targeted therapy with small molecule inhibitors of fms-like thyrosine kinase 3 (midostaurin, gilteritinib), dehydrogenase isocitrate 1 (AG-221), and dehydrogenase isocitrate 2 (AG-120) to the treatment of R/R AML are excellent examples [37,38,39,40]. Upon the absence of actionable mutations, the primary focus would be on non-chemotherapeutic strategies for the treatment of R/R AML [33]. One of them are drugs targeting the anti-apoptotic BCL2 family proteins [41,42].

In our previous studies, we demonstrated a number of genes which were overexpressed in AML, including *STMN1*, *NPM1*, *CAT*, and *WT1* [43,44]. We also showed that at the time of AML diagnosis, high *NPM1* expression correlated with poor outcome [44] and the *WT1* expression increased with treatment resistance [43]. Due to an increasing interest in the role of pro-apoptotic drugs in overcoming leukemic cell resistance, the main aims of this study were to analyze the expression pattern of *BCL2* family genes in AML patients and associate it with the retrospectively monitored response to therapy, as well as disease-free and overall survival. Looking for factors influencing the BCL2 family expression in AML, we also analyzed the expression of genes encoding proteins interacting with BCL2 and checked their mutational status. To do so, we took advantage of the RNA-seq and exome sequencing data collected in our laboratory.

## 2. Materials and Methods

Samples. Peripheral blood (PB) or bone marrow (BM) samples were collected in 2007–2010 from 27 adult patients with AML, M1 or M2 FAB, at the time of the first diagnosis (Table 1, Table S1). Control samples were collected from healthy volunteers (one BM and 12 PB samples) and from an AML patient after treatment (in complete remission stage, one BM sample). Patients were diagnosed and treated at the Department of Hematology and Bone Marrow Transplantation in the University Hospital of the Lord’s Transfiguration of Poznan University of Medical Sciences in Poznan, Poland. In the case of all AML patients, induction therapy was applied according to the following scheme: daunorubicin 60 mg/m^2^ per day given as a 1–3 h-long intravenous infusion on days 1–3 and cytosine arabinoside 200 mg/m^2^ per day as a continuous intravenous infusion on days 1–7 (3 + 7 regimen). Complete remission (CR) in AML was defined using the criteria developed by an International Working Group [45,46,47], including: (1) normal values for absolute neutrophil count (>1000/µL) and platelet count (>100,000/µL), and independence from red cell transfusion; (2) no clusters or collections of blast cells in bone marrow biopsy and no symptoms of extramedullary leukemia (e.g., the central nervous system or soft tissue involvement); (3) normal maturation of all cellular components (i.e., erythrocytic, granulocytic, and megakaryocytic series) in the bone marrow aspiration biopsy; (4) less than 5% percent blast cells in the bone marrow, and no blast cells expressing leukemic phenotype (e.g., Auer rods, aberrant immunophenotype blasts). The “primary refractory disease” (RES, resistant to treatment) was diagnosed when failure of only one induction cycle was confirmed [48].

RNA-seq. PB or BM mononuclear cells were separated using density gradient centrifugation (Gradisol L, Aqua-Medica, Poland). Total RNA was extracted with a mirVana miRNA Isolation Kit (Ambion/Thermo Fisher Scientific, Waltham, MA, USA) and DNase-treated (TURBO DNA-free kit, Ambion/Thermo Fisher Scientific). RNA-seq was performed with a Genome Analyzer IIx (Illumina, San Diego, CA, USA). Up to 4 µg of total RNA was used to prepare sequencing libraries with the TruSeq RNA Sample Prep Kit (Illumina). The indexed libraries were sequenced on a single-read flow cell (TruSeq SR Cluster Kit v2 cBot, Illumina), two libraries per lane, with 72-nt long reads. The data were processed by STAR-aligner v. 2.5.3a (https://github.com/alexdobin/STAR, accessed on 15 November 2018, Alexander Dobin, Cold Spring Harbor Laboratory, New York, NY, USA) [49] for mapping, and RSEM ver. 1.3.0 (https://github.com/deweylab/RSEM, accessed on 14 February 2018, Dewey Lab, University of Wisconsin-Madison, Madison, WI, USA) [50] for gene quantification. The levels of expression were normalized first for the gene length and, subsequently, for the sequencing depth in order to obtain TPM (transcripts per million) values.

Statistical analysis. Statistical analyses were made in R ver. 4.0.4 (https://cran.r-project.org, accessed on 27 March 2021, R Foundation for Statistical Computing, Vienna, Austria) and R Studio ver. 1.4.1106 (https://www.rstudio.com, accessed on 27 March 2021, RStudio, Boston, MA, USA). The following R packages were used: base, DESeq2 [51], ggplot2, ggcorrplot, plyr, reshape2, ggsignif, ggpubr, Hmisc, and survival. Plots were generated with R or Microsoft Excel. To compare the levels of gene expression between AML and additional control samples from Human Protein Atlas, Mann-Whitney test with Bonferroni correction for multiple comparisons was used. To estimate the patient outcome and correlate it with particular gene expression levels, Kaplan-Meier analysis was applied. The differences between survival curves were tested with a log-rank test. For the association of gene expression with mutation status and clinical features, ANOVA and MANOVA tests were applied, for univariate and multivariate analysis of variance, respectively.

Mutation analysis. Whole exome sequencing (WES) data collected for the analyzed AML samples in our laboratory were used to extract information about mutational status of the genes of interest (26 BCL2 family members, 16 genes encoding BCL2-interacting proteins and 12 genes recurrently mutated in AML). Haplotype calling was made with GATK v. 4.0.12.0, (https://gatk.broadinstitute.org/, accessed on 2 February 2019) [52]. GATK-originated vcf files, generated for each sample separately, were analyzed with the use of eVai v. 0.6 (enGenome, www.engenome.com/evai/, accessed on 24 November 2019), with the application of the following filters: effect (all splice and coding variants, except for synonymous, were selected); sample (coverage ≥ 6; quality ≥ 40; allele frequency ≥ 0.1), and classification (only pathogenic, likely pathogenic and uncertain variants were maintained). Alternative allele coverage was used as an additional threshold (≥3 for SNPs and ≥4 for indels).

## 3. Results

### 3.1. Expression of Genes Encoding Proteins from the BCL2 Family

The list of 25 genes encoding proteins from the BCL2 family (Table S2) was downloaded from the HUGO Gene Nomenclature Committee (HGNC) repository (https://www.genenames.org/data/genegroup/#!/group/1057 accessed on 4 March 2021). Based on the differences in the protein structure described above, 14 genes are classified as the “BCL2 family” and the remaining 11 as the “BCL2 homology region 3 (BH3) only”. We decided to add also one fusion transcript (*BCL2L2-PABPN1*) to the list, which naturally occurs as a product of read-through transcription of *BCL2L2* and its neighboring gene, *PABPN1*. This chimeric RNA was shown to be overexpressed in bladder cancer [53].

**Figure 1:**
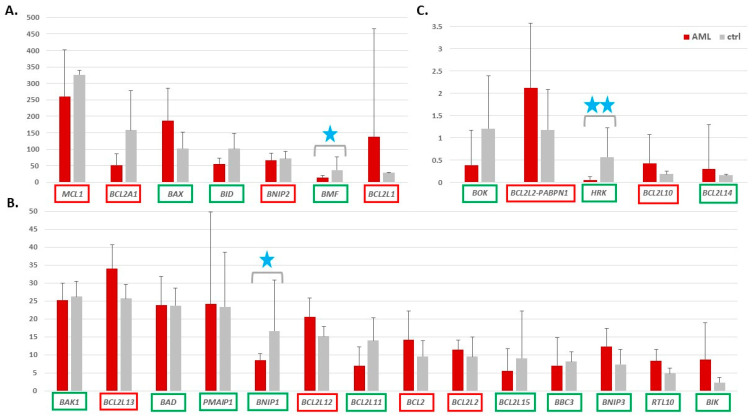
Expression levels of 26 genes from the *BCL2* family in AML versus control (ctrl) samples. The expression levels were extracted from the RNA-seq data and presented as mean TPM (transcripts per million) values with standard deviation bars (*y* axis). For clarity, the genes were ranked according to the TPM in control samples and divided into three separate graphs, from (**A**) the highest to (**B**) middle and (**C**) the lowest gene expression. The genes encoding the pro-apoptotic proteins are indicated in green, the anti-apoptotic are in red frames. Blue stars indicate genes with statistically significant expression change between AML and ctrl (adj. *p* value threshold 0.05; 1 star < 0.01; 2 stars < 0.001). The statistical data were calculated with DESeq2, which was applied for differential analysis of the whole RNA-seq data set, with a default method for multiple testing correction.

The normalized expression values (TPM, transcripts per million) for the 26 *BCL2* family genes mentioned above were extracted from RNA-seq data generated in our laboratory for 27 AML M1 and M2 patients (Table 1, Table S1), from BM or PB samples collected at the time of AML diagnosis. Control samples (ctrl) were represented by three specimens: (i) a BM sample from a healthy volunteer; (ii) a pool of 12 PB samples delivered by healthy volunteers; (iii) a BM sample from an AML patient after treatment (in complete remission stage). Mean TPMs of all 26 genes in AML and control samples are presented in Figure 1, whereas the source data (TPM values for individual samples) are available in Table S3. The statistical data was extracted from the results of differential expression analysis, generated for the whole RNA-seq data set with the use of DESeq2 (Table S3).

Only five genes, namely *MCL1*, *BAX*, *BCL2A1*, *BCL2L1*, and *BID*, revealed high expression levels (TPM > 100) in the AML or ctrl samples (Figure 1A). The expression of five other genes was barely detectable (TPM about 2 and below, Figure 1C) whereas the majority of the BCL2 family members showed low to middle expression level (TPM range 5–35), including the *BCL2* itself with mean TPM 14 in AML and 10 in ctrl (Figure 1B). About half of pro-apoptotic genes (Figure 1, names in the green frames) revealed decreased expression trend in AML, as compared to ctrl, and for three of them, *BMF*, *BNIP1*, and *HRK*, the expression difference between AML and ctrl was statistically significant (adj. *p* value < 0.01) (Figure 1, blue stars).

The majority of the anti-apoptotic genes showed an upregulation trend in AML and only one (*BCL2A1*) was decreased in AML vs. ctrl but no difference was statistically significant. The reason for it might be high variability between both, AML patients and control samples, reflected by high standard deviation values.

Because the number of control samples was not high in our dataset, we extracted control RNA-level data (mean TPM for 6 PB mononuclear cell samples and mean TPM for 4 BM samples) from the Human Protein Atlas (HPA, www.proteinatlas.org accessed 5 June 2021, Table S4A) and repeated the above analysis including additional controls. Each gene expression was compared between AML and control with the use of Mann-Whitney test. The results were comparable with the results of previous analysis but only the increase of *BCL2L13* in AML was statistically significant when Bonferroni correction for multiple comparisons was applied (Figure S1, Table S4B). The levels of BCL2 family transcripts in our controls were highly correlated with the levels of HPA transcripts (Spearman correlation coefficient 0.85 for PB and 0.93 for BM, Table S4C).

**Figure 2:**
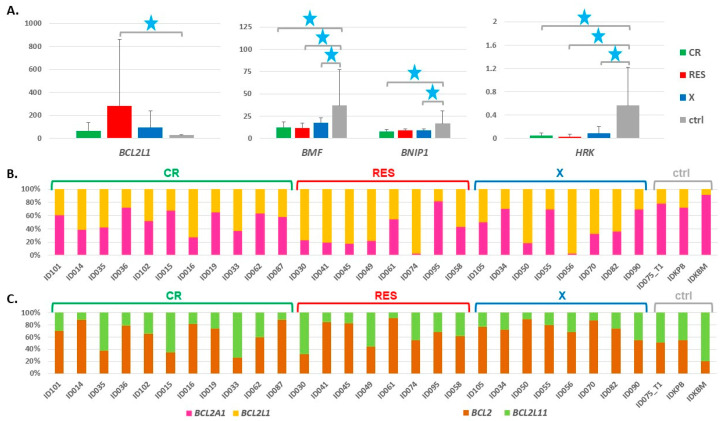
(**A**) Expression levels of four genes from the *BCL2* family in AML and ctrl samples, with AML divided into three groups according to patient response to the first induction therapy (CR—complete remission; RES—resistance to therapy; X—death during therapy). The expression levels were extracted from the RNA-seq data collected at the time of diagnosis (before treatment) and presented as mean TPM (transcripts per million) values with standard deviation bars (*y* axis). Blue stars indicate statistically significant expression changes (adj. *p* value threshold 0.05; 1 star < 0.05). The statistical data was calculated with DESeq2, which was applied for differential analysis of the whole RNA-seq data set, with a default method for multiple testing correction. (**B**,**C**) Ratio of the expression of two pairs of genes: *BCL2A1* and *BCL2L1* (**B**); *BCL2* and *BCL2L11* (**C**) in individual AML and ctrl samples, presented as a percentage contribution of each gene into the sum of TPMs of both compared genes.

To retrospectively analyze the association between the *BCL2* gene family expression at the time of AML diagnosis and the patient response to therapy monitored at a later stage, we divided AML into three groups: CR—patients who reached complete remission after the first induction therapy (*n* = 11); RES—patients resistant to the first induction therapy (*n* = 8); X—patients who succumbed to the disease during therapy (*n* = 8) (Table 1). The results are summarized in Table S3 (all 26 genes) and Figure 2A. Three genes (*BMF*, *BNIP1*, and *HRK*) were significantly decreased in two or three AML groups, as compared to ctrl. *BCL2L1* was the only gene increased in AML patients resistant to therapy. The difference was statistically significant in a comparison between RES and ctrl (adj. *p* value 0.03). In the RES group, *BCL2L1* revealed the highest level of expression among all the *BCL2* family members (mean TPM 283). The variability of this anti-apoptotic gene expression within the RES group was also extremely high (TPM range 18–1702, Table S3).

Comparing the BCL2 family members, we noticed similar expression levels but inverse relation between AML and ctrl for *BCL2A1* and *BCL2L1* genes. Looking at individual samples, we found that *BCL2L1* remarkably predominated over *BCL2A1* in AML, in particular in RES (Figure 2B). The difference in *BCL2A1/BCL2L1* ratio was statistically significant not only between AML (as a whole group or divided into CR, RES and X) and ctrl, but also between RES and CR (Table 2). Another interesting relation was observed between *BCL2* and *BCL2L11* gene, which encodes one of the BCL2 antagonists, BIM [54]. The levels of these two genes were balanced in ctrl or shifted towards the pro-apoptotic *BCL2L11*. On the contrary, in the majority of the AML samples, including all from the X subgroup, the pro-apoptotic *BCL2* evidently predominated (Figure 2C). The difference in *BCL2/BCL2L11* ratio was statistically significant between AML and ctrl (*p* = 0.033) and between X and ctrl (*p* = 0.012) (Table 2).

A correlation analysis of the *BCL2* family gene expression showed differences in the correlation pattern not only between AML and ctrl, but also between the CR, RES, and X AML subgroups (Figures S2 and S3), which suggests that the balance between the expression levels of particular family members is very delicate and dynamic, and can be easily disturbed. Only some correlations were statistically significant, e.g., in AML we observed a positive correlation between *BCL2L1*, *BOK*, and *BCL2L11*, a negative correlation between *BCL2L1* and *BID*, a positive correlation between *BAX*, *BBC3*, *PMAIP*, positive correlation between *BCL2*, *BMF*, and *BNIP3*, a negative correlation between *BCL2A1* and *RTL10*, or a negative correlation between *BCL2A13* and *BCL2A14* (Figure S2D). Strikingly, positive as well as negative correlations were observed between genes encoding both the pro- and anti-apoptotic proteins.

### 3.2. Expression of the BCL2L1 Gene Isoforms

**Figure 3:**
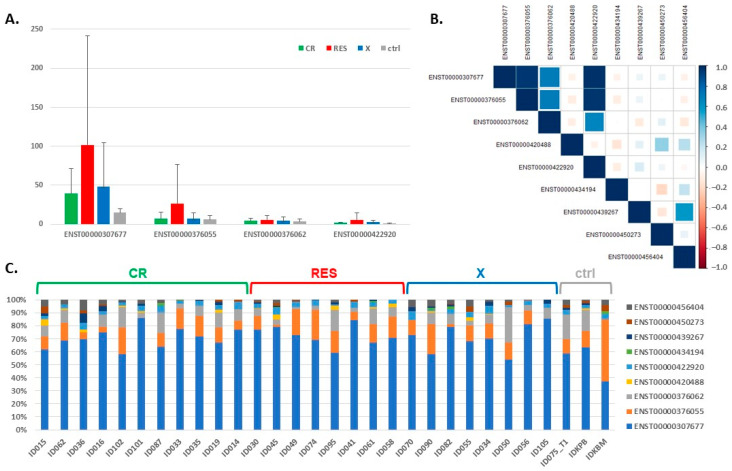
The expression of the *BCL2L1* gene isoforms, extracted from the RNA-seq data. (**A**) The expression levels of four dominant isoforms in AML and control samples, with AML divided into three groups with different response to therapy; (**B**) the graphical presentation of Spearman correlation of the expression calculated for each pair of the *BCL2L1* gene isoforms detected by the RNA-seq. The color intensity and size of the squares are proportional to the correlation coefficients. (**C**) The contribution of particular *BCL2L1* isoforms to the total expression of the *BCL2L1* gene in AML and control samples.

Because *BCL2L1* was the only gene specifically increased in RES and its function depends on the type of isoform generated as a result of alternative splicing [55], we compared the levels of expression of the *BCL2L1* gene isoforms extracted from the RNA-seq data. Among nine isoforms identified, the expression of five was barely detectable (TPM ≤ 1, Table 3). Four isoforms with the highest expression level are presented in Figure 3A. Two of them, ENST00000307677 and ENST00000376062, encode a longer protein (BCL-XL, 233 aa), which acts as an apoptotic inhibitor, while the remaining two—a shorter one (BCL-XS, 170 aa)—acts as an apoptotic activator [55]. The expression of these four isoforms was highly correlated in individual patients (Spearman correlation coefficients 0.7–0.98) (Figure 3B). Isoform ENST00000307677, encoding an apoptosis inhibitor, contributed the most to the total expression of the *BCL2L1* gene (Figure 3C). In AML, it was ~60–80%, with a mean of 71.3% (70.5% in CR, 72.4% in RES and 71.2% in the X group) (Table S5). In the control samples, this isoform was found in lower proportions (~40–60%, mean 53.2%), and the difference in the ENST00000307677 contribution to the total *BCL2L1* expression was statistically significant between RES and ctrl, as well as between CR and ctrl (Wilcoxon rank sum exact test, *p* = 0.018 and *p* = 0.049, respectively).

### 3.3. Expression of Genes Encoding Proteins Interacting with BCL2L1

**Figure 4:**
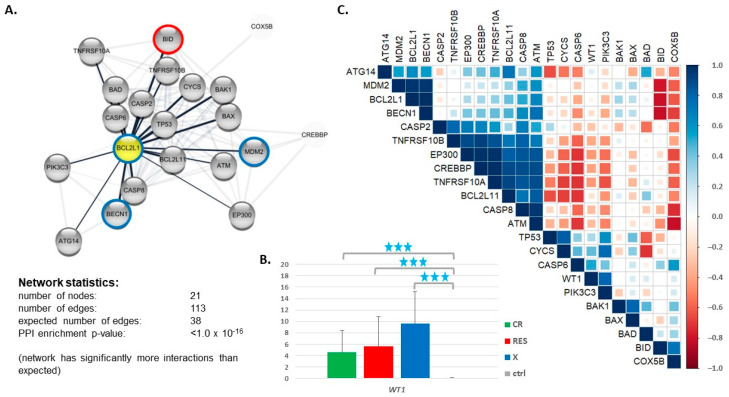
The expression of genes encoding proteins interacting with BCL2L1. (**A**) The network of 20 proteins interacting with BCL2L1 generated by STRING (https://string-db.org accessed on 7 March 2021); (**B**) *WT1* gene expression in AML (divided into CR, RES and X groups) and ctrl, extracted from the RNA-seq data; (**C**) a graphical presentation of Spearman correlation of expression calculated for each pair of the genes encoding proteins interacting with BCL2L1, based on the RNA-seq data collected for RES AML patients. The color intensity and size of the squares are proportional to the correlation coefficients. The colored circles on the plot (**A**) indicate proteins encoded by the genes whose expression was significantly (*p* > 0.05) correlated with the expression of *BCL2L1*, blue circles mean a positive correlation and red—a negative one.

Looking for other factors which, in association with *BCL2L1*, may potentially contribute to AML therapy failure, we applied the STRING protein interaction database (https://string-db.org www.proteinatlas.org accessed on 7 March 2021) to generate a network of 20 proteins interacting with BCL2L1 (Figure 4A). The network included the BCL2 family members (BCL2L11, BAD, BAK1, BAX, BID) and other proteins engaged in the regulation of apoptosis and tumor development: caspases (CASP2, CASP6, CASP8); Tumor Protein P53 (TP53); tumor necrosis factor (TNF) receptor superfamily members (TNFRSF10A, TNFRSF10B); cytochromes (CYCS, COX5B); kinases (PIK3C3—Phosphatidylinositol 3-Kinase Catalytic Subunit Type 3 and ATM Serine/Threonine Kinase); autophagy related proteins (ATG14 and BECN1 (beclin 1)); transcription regulators with histone acetyltransferase activity (EP300 (E1A Binding Protein P300) and CREBBP (CREB Binding Protein)); and MDM2 Proto-Oncogene. Based on the literature data, we decided to also include the Wilms Tumor Protein (WT1), which was described as a mediator of TRAIL (tumor necrosis factor-related apoptosis-inducing ligand) resistance in AML by regulating BCL2L1 [56]. However, according to STRING, WT1 does not interact directly with BCL2L1, but through TP53. The RNA-seq data revealed that the *WT1* gene was highly upregulated in AML vs. ctrl, and the level of the *WT1* expression presented an increasing trend from CR to X (Figure 4B), which confirm our earlier microarray-based results [43]. Any other gene from the BCL2L1-interacting network described above was differentially expressed in our dataset (Table S2).

Correlation analysis of gene expression, performed exclusively for the RES group (Figure 4C, Table S6), revealed a cluster of strongly positively correlated genes, including *BCL2L11*, *EP300*, *CREBBP*, *CASP8*, *ATM*, *TNFRSF10A*, and *TNFRSF10B*. These genes were strongly negatively correlated with *CASP6*. *BCL2L1* was highly positively correlated with two genes, namely *BECN1* and proto-oncogene *MDM2*, and negatively correlated with *BID*. *WT1* revealed a significant positive correlation only with *PIK3C3*. Other statistically significant positive correlations were noted for the following gene pairs: *CASP2* & *TNFRSF10B*; *TP53* & *CYCS*; *CYCS* & *PIK3C3*; *BECN1* & *ATM*; *BCL2L11* & *ATG14*; *BAK1* & *BAX*, *BID* & *COX5B*. A statistically significant negative correlation was also found for *CYCS* & *BAD*.

### 3.4. Impact of the BCL2 Gene Family Expression Levels on the Patient Outcome

**Figure 5:**
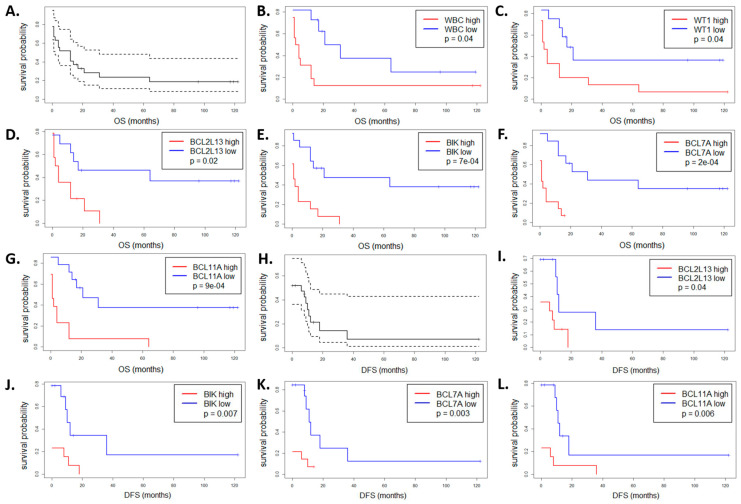
Overall survival (OS, **A**–**G**) and disease-free survival (DFS, **H**–**L**) of 27 AML patients as a whole group (**A**,**H**) and divided according to the WBC count (**B**) or the expression level of the following genes: *WT1* (**C**); *BCL2L13* (**D**,**I**); *BIK* (**E**,**J**); *BCL7A* (**F**,**K**); *BCL11A* (**G**,**L**). In each case, the blue curve means low level and the red curve high level of expression. Only the genes with statistically significant differences between the two curves are shown (the log-rank test *p* value threshold 0.05).

To associate the expression of genes from the BCL2 family, measured at the time of AML diagnosis, with disease prognosis, we performed a survival analysis with the use of Kaplan-Meier estimator. This type of retrospective analysis did not take into account patient response to the first induction therapy, but the time of disease-free and overall survival, which was monitored for up to 10 years after the first AML diagnosis. In the analyzed group of 27 AML patients, the median disease-free survival (DFS) and median overall survival (OS) were equal to 0 and 12 months, respectively (Figure 5A,H). As expected, high WBC count was associated with shorter OS and DFS (*p* = 0.04, Figure 5B and Figure S4F). Similarly, high level of the *WT1* expression was associated with shorter OS (*p* = 0.04, Figure 5C).

To analyze the impact of the *BCL2* family gene expression levels on DFS and OS, we dichotomized samples based on the median level of a particular gene. The results indicated that the expression of seven genes may be associated with patient outcome, as presented in Figure 5 and Figure S4. In all cases except one (*BCL2L1*), high gene expression at the time of AML diagnosis was associated with worse prognosis. However, this effect was statistically significant for only two genes, namely *BCL2L13* (*p* = 0.02 for OS and 0.04 for DFS, Figure 5D,I) and *BIK* (*p* = 7× 10^−4^ for OS and 0.007 for DFS, Figure 5E,J).

In addition, we performed a similar analysis for the members of other BCL families and found two genes whose expression levels were significantly associated with the patient outcome: *BCL7A* (*p* = 2× 10^−4^ for OS and 0.003 for DFS, Figure 5F,K) and *BCL11A* (*p* = 9 × 10^−4^ for OS and 0.006 for DFS, Figure 5G,L). They encode BAF chromatin remodeling complex subunits and are involved in lymphoma pathogenesis. Similarly as in the case of the BCL2 family members, high expression of these genes in AML at the time of diagnosis was associated with poor outcome.

### 3.5. Mutation Analysis of the BCL2 Family Genes and Genes Encoding Proteins Interacting with BCL2L1

**Figure 6:**
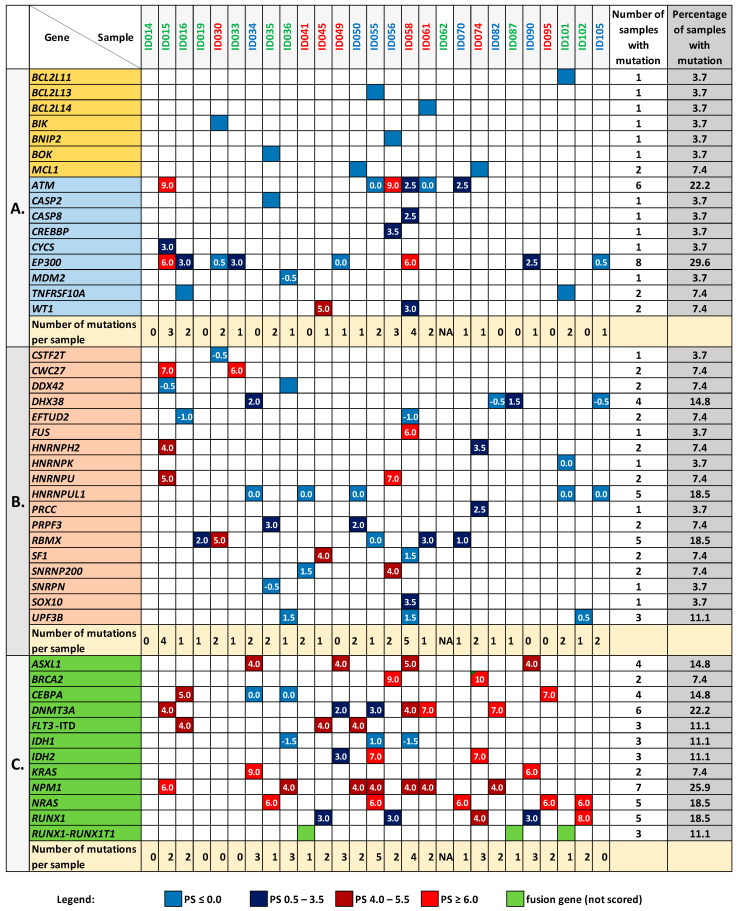
The mutation patterns in the studied genes in the samples collected from patients at the time of AML diagnosis. (**A**) mutations in the *BCL2* family members (yellow background) and genes encoding proteins interacting with BCL2L1 (blue background); (**B**) mutations in the genes encoding splicing-related proteins; (**C**) mutations in other genes, which are recurrently mutated in AML. The presence of a mutation is indicated by a colored square. The numbers inside squares reflect the pathogenicity scores (PS) estimated by eVai software (the higher PS, the more significant the mutation). If more than one mutation was detected in a sample, the highest PS value is presented. The lack of a number means no PS score assigned to a variant (PS NA). In panel (**A**), all genes with a mutation in at least one sample are shown, whereas in panels (**B**,**C**), only genes with PS-assigned mutations are presented. Tables S7 and S8 contain full lists of variants detected in the studied genes and analyzed samples.

Taking advantage of exome sequencing, we explored the data collected in our laboratory for the analyzed sample set and checked whether any noteworthy mutation occurred in the *BCL2* family genes and genes encoding proteins interacting with BCL2L1. The analysis was made with the use of eVai software (enGenome) and included only coding (except for synonymous) and splice variants that met the quality criteria described in the Methods section. Among the 26 BCL2 family members tested, we found single mutations in 7 genes: *BCL2L11*, *BCL2L13*, *BCL2L14*, *BIK*, *BNIP2*, *BOK*, and *MCL1* (Figure 6A). *MCL1* was the only gene mutated in two AML samples (ID050 and ID074), and in both cases, it was the same missense mutation (*MCL1*:c.680C>T; *p*.Ala227Val). This mutation, reported in the dbSNP database (rs11580946) with AF < 0.84% (GnomAD_exome), was classified by eVai software as a variant with uncertain significance (PaPI score: 0.8 (DAMAGING); PolyPhen-2: 0.303 (TOLERATED); DANN score: 0.99; SIFT: 0.02 (DAMAGING)). None of the mutations detected in the *BCL2* family genes was classified as pathogenic, and none was assigned with any pathogenicity score (PS), which is a measure calculated by eVai based on a set of implemented variant classification criteria (Figure 6A, Table S7).

With respect to the genes encoding proteins interacting with BCL2L1, 9 out of the 16 analyzed genes were mutated in at least one AML sample. In total, 35 different mutations were detected in these genes, including three which occurred in two samples. In contrast to the *BCL2* family analysis, PS was not determined for only three variants here (Figure 6A, Table S7). The mutations in *EP300* and *ATM* genes were the most significant (PS up to 6–9) and overrepresented, being detected in 8 (29.6%) and 6 (22.2%) samples, respectively. Mutations in *WT1* and *TNFRSF10A* were present in two samples and the remaining genes (*CASP2*, *CASP8*, *CREBBP*, *CYCS*, *MDM2*) were mutated in only one sample. We identified no noteworthy mutation neither in *ATG14*, *BECN1*, *CASP6*, *COX5B*, *PIK3C3*, *TNFRSF10B* nor in *TP53*.

Notably, in one-third of AML samples (9 out of the 27 analyzed), we found no significant mutations neither in the *BCL2* family members, nor in the genes encoding proteins interacting with them, and in another set of nine samples, only one mutation was noted (Figure 6A). The list of all the identified mutations is available in Table S7.

### 3.6. Mutation Analysis in the Genes Encoding Splicing-Associated Proteins

Dysregulation of proteins involved in apoptosis and oncogenesis may also be a consequence of splicing disruption, triggered by an uncontrolled overexpression of splicing factors or mutation of the genes which encode them. Therefore, we analyzed the mutational status of 188 genes implicated in the splicing. The list of genes was downloaded from the mRNA Splicing—Major Pathway (https://pathcards.genecards.org/Pathway/2004 accessed on 24 March 2021). Approximately half of the genes (98) were mutated in at least one AML sample and there was no sample without a mutation in the splicing-related genes (Figure S5). In total, 213 unique mutations were identified (Table S8); however, only 2 were pathogenic (frameshift variants in *HNRNPU* and *CWC27* genes) and 3 were likely pathogenic (in *CWC27*, *FUS* and *RBMX* genes). 179 mutations had no PS at all. Figure 6B presents all the PS-attributed mutations which were identified in the 18 splicing-related genes. The most frequently mutated genes were *RBMX* and *HNRNPUL1* (mutations detected in 5 samples), *DHX38* (mutations detected in 4 samples), and *UPF3B* (mutations detected in 3 samples).

### 3.7. Relations between the BCL2 Family Gene Expression, Mutation Status, and Other Clinical Features

To associate the *BCL2* family gene expression with the presence of particular mutations and other clinical features, we applied two types of analysis of variance. Only the mutations which occurred in at least two samples were taken into account. Apart from the mutations described above, we included also the mutations detected in the 12 genes recurrently mutated in AML (Figure 6C) in the analysis. A univariate analysis with two-way ANOVA revealed a total of 81 statistically significant (*p* value < 0.05) pair-wise relations between the expression levels of 25 BCL2 family members, the mutational status of 26 genes, and the following clinical features: AML FAB subtype, karyotype, tissue type, WBC count, the percentage of blasts in PB and BM, response to treatment (Table S9A).

MANOVA which included expression of all the *BCL2* family members in one test, found a statistically significant relation to age (*p* = 0.02372) and mutational status of two genes, *EP300* (*p* = 0.03293) and *DDX42* (*p* = 0.006992) (Table S9B). Other statistically significant associations are presented in Table 4. The most significant relation was found between the expression of four genes (*BCL2L1*, *BID*, *BOK*, *HRK*) and the mutation status of two genes: *BRCA2* (*p* = 4.145 × 10^−10^) and *RUNX1* (*p* = 0.04287) (Figures S6 and S7). The overexpression of *BOK*, *BCL2L1*, *BCL2L11*, and *MCL1* was also associated with the presence of the *IDH2* mutation (*p* = 0.01203). It is not surprising, as the expression of three genes overrepresented in the above-mentioned tests, i.e., *BCL2L1*, *BCL2L11* and *BOK*, was highly correlated in AML (Figure S2B). Notably, one sample (ID074) with an extremely high level of *BCL2L1*, was collected from a PB of a patient presenting mutations in *BRCA2*, *IDH2*, and *RUNX1*, and the lowest number of WBC in the whole group of the AML patients. Regarding single gene impact, the *BCL2L1* expression was significantly associated only with the presence of the *BRCA2* and *IDH2* mutation (*p* = 1.179× 10^−7^ and 0.01512, respectively), the *BCL2L11* expression with the *IDH2* mutational status (*p* = 0.02728), the WBC count (0.00866) and the tissue type (0.01831), whereas the *BOK* expression—with the *BRCA2* status (*p* = 6.211 × 10^−5^), the tissue type (0.0004571) and the *IDH2* status (*p* = 0.0012835).

**Figure 7:**
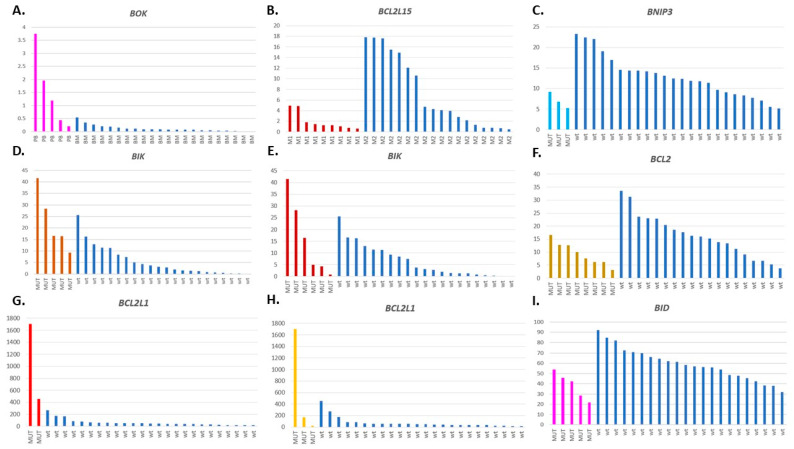
The expression patterns of selected *BCL2* family genes in relation to (**A**) tissue type (PB or BM), (**B**) AML FAB type (M1 or M2), and mutation status of the following genes: (**C**) *RUNX1-RUNX1T1* (fusion gene resulting from t(8;21)); (**D**) *RBMX*; (**E**) *ATM*; (**F**) *EP300*; (**G**) *BRCA2*; (**H**) *IDH2*; (**I**) *RUNX1*. On panels (**A**,**D**,**G**), the first sample from to the left is ID074.

Other statistically significant associations were found between the expression of gene groups and FAB type or the presence of mutations in genes recurrently mutated in AML (*NPM1*, *RUNX1*, *CEBPA*, *DNMT3A*, *KRAS*), genes encoding proteins interacting with the BCL2 family (*ATM*, *EP300*) and genes encoding splicing-related proteins (*RBMX*, *HNRNPU*, *HNRNPH2*, *HNRNPUL1*, *SNRNP200*, *DDX42*, *DHX38*, *CWC27*) (Table 4). The results of all the tests performed are presented in Table S9. The selected examples are graphically presented in Figure 7.

## 4. Discussion

Apoptosis is a physiological process involved in the development of a multicellular organism and tissue renewal, e.g., hematopoiesis [10,57]. Relative levels of the BCL2 family members are critical in fine-tuning a cell’s fate [15,16,58] and change depending on the cell type and the stage of differentiation [59,60]. Although enhancing cell survival by BCL2 proteins usually does not affect cell proliferation and differentiation [61,62,63,64], a disturbed balance between pro-apoptotic and anti-apoptotic proteins often leads to tumorigenesis [8]. While excessive proliferation and blockade of differentiation are essential in the development of AML, inhibition of apoptosis is not negligible, particularly in refractory disease. Indeed, high *BCL2* expression in the AML cells was detected at presentation, disease relapse, and treatment resistance [65,66]. To date, the BCL2 family research in AML has focused mainly on *BCL2*, and, though to a lesser extent, on *MCL1* and *BCL2L1.* Nagy et al. [67] found that *BCL2*, *BCL2A1*, *MCL1*, and *BAX* were highly expressed in leukemia and lymphoma patients. Compared to healthy controls, *BCL2L1* was underexpressed in chronic leukemias, but upregulated in mantle cell lymphoma and in some acute leukemia patients [67]. Here, we show that a dysregulation of the expression in AML concerns many other, although not all, BCL2 family members. Despite the general trend of upregulation of anti-apoptotic and downregulation of pro-apoptotic genes in AML, as compared to healthy controls, only a decrease in the genes encoding three pro-apoptotic proteins, i.e., *BMF*, *BNIP1* and *HRK*, was statistically significant. This indicates the dominant role of the BH3-only-domain proteins in the apoptosis inhibition in AML. The main function of these proteins is activating BAX and BAX-like pro-apoptotic family members, and sequestering anti-apoptotic antagonists by forming heterodimers [68]. Therefore, insufficiency of BH3-only-domain proteins makes anti-apoptotic factors free and ready to act.

Comparing relative expression levels of particular family members, we found that in AML, the level of only three genes, *MCL1*, *BAX*, and *BCL2L1*, was high. *MCL1* encodes one of the most essential proteins of the BCL2 family and its overexpression was associated with improved survival of hematopoietic stem cells in transgenic mice and immortalization of myeloid cell lineages [64]. Because BCL-XL and MCL-1 proteins appear to have complementary functions, acting by sequestering BAK [69], it is possible that the increased *BCL2L1* observed here replaced the insufficiently expressed *MCL1* to maintain anti-apoptotic activity in leukemic cells. Both genes revealed much higher expression than the expression of the *BCL2* gene, the best known family member with an anti-apoptotic activity, commonly associated with drug resistance and considered a therapeutic target. The importance of BCL-XL in AML was perceived by Pallis et al. [70] who detected high levels of this protein in AML blasts with autonomous growth in vitro and associated it with poor response to therapy. Similarly, in our study, the *BCL2L1* expression was increased in patients resistant to induction therapy, though it was highly variable between patients. This is consistent with the results of Kaufmann et al. [71] who found that protein levels of BCL2, MCL-1, BCL-XL, and BAX, measured at the time of AML or ALL diagnosis, varied over as much as a 40-fold range between individual patients.

*BCL2L1* upregulation was found in different tumors [72,73,74,75,76,77]. In the study of a large cohort of colorectal cancers, BCL-XL, but not BCL2 or MCL-1, emerged as a highly active protein, and its transcript level was the highest among the anti-apoptotic *BCL2* genes [77].

Functional studies of BCL2 family are impeded by the fact that the loss of Bcl-XL or Mcl-1 causes embryonic lethality in mice and the loss of Bcl2 reduces mice lifespan [78,79]. Bcl2a1 locus in mice is quadruplicated, which does not make it a perfect model in gene targeting studies, and the physiological role of this protein is still not completely understood. *BCL2A1*, regulated by the NF-kB transcription factor, is mainly expressed in the hematopoietic system, facilitating survival of leukocytes during their maturation and differentiation [80]. Here, *BCL2A1* was the only anti-apoptotic family member, which was decreased in the majority of the AML samples. The lack of statistical significance was probably due to the high difference in *BCL2A1* level between control PB and BM samples, reflected also in HPA data. In AML, *BCL2A1* expression was much lower than in healthy BM but similar to that detected in healthy PB. The *BCL2A1* decrease was accompanied by a decrease of the *BID* gene, encoding a BH3-only protein described as the BCL2A1 partner [80]. In accordance with the literature data, the expression of *BCL2A1* was also highly correlated with the expression of two transcription factors, namely *NFKB1* and *NFKBIA* (Figure S8).

Dysregulation of gene expression may also have a genetic background. Beroukhim et al. [81], studying genome-wide somatic copy-number alterations in a large collection of cancers, found frequent amplifications of the regions surrounding two anti-apoptotic genes, *MCL1* and *BCL2L1*, and deletions containing two pro-apoptotic *BCL2* family members, *BOK* and *BBC3*. Unfortunately, whole genome sequences of the AML patients studied here are not available to compare it. From the analysis of exome sequencing data, we concluded that the *BCL2* family genes were rather not mutated in AML. Similarly, in the literature, small somatic mutations in the *BCL2* family are noted occasionally [82,83,84,85,86,87]. Moreover, we did not detect mutations in the *TP53* gene, encoding the tumor suppressor p53 protein, a regulator of cell cycle arrest or apoptosis upon DNA damage and cellular stress. In the studied samples, the mutations in genes recurrently mutated in AML, e.g., *NPM1*, *DNMT3A*, *NRAS*, *RUNX1*, *ASXL1*, *ATM*, *CEBPA* seemed to play a pivotal role in leukemogenesis, whereas changes in the *BCL2* family gene expression probably played a secondary role. Recently, Bilbao-Sieyro et al. [88] reported a positive association between the absence of the *NPM1* mutation and a higher *BCL2* level. In our data, four other genes (pro-apoptotic *BID*, *BIK*, *BNIP1*, and *BCL2L13* encoding a protein of controversial function) presented significantly higher expression in the *NPM1*-mutated samples. In consistence with our results, Gaidzik et al. [89] reported dysregulation of the apoptotic pathway, supported in particular by an increase in *BCL2L1* expression, as a key feature of *RUNX1*-mutated AML. The presence of mutation in *RUNX1* gene, encoding one of a crucial regulators of myelopoiesis, was associated by the authors with the presence of *IDH1/IDH2* mutations and resistance to induction chemotherapy. In our data, the expression of three *BCL2* family members (*BCL2L1*, *BID* and *HRK*) was correlated with *RUNX1* mutation. *BCL2L1* and *HRK* were upregulated whereas *BID*, encoding a pro-apoptotic BH3-only protein, was downregulated in *RUNX1*-mutated samples. *BID* inversely correlated with the level of antiapoptotic *BCL2L1* in AML, in particular in RES. This suggests that *RUNX1*-mutation-driven apoptosis dysregulation is mainly based on increase of *BCL2L1* and decrease of *BID* and both events contribute to treatment resistance. Other authors demonstrated that *IDH1*- and *IDH2*-mutated AML cells were highly dependent on the expression of *BCL2* for their survival [90].

Furthermore, in line with our findings, Salmon et al. [91] depicted dysregulation of apoptosis-related genes as a possible consequence of the *BRCA1*- and *BRCA2*- mutations in breast cancer. Although solid tumors cells are believed to be more dependent on BCL-XL than hematologic malignancies, our results suggest that some AML cases, for example, *BRCA2*- or *IDH2*-mutated, may need this particular protein more than the other BCL2 family members for their survival. However, further research is advisable due to a small set of mutated samples studied here.

Another factor which may influence the *BCL2* family gene expression is splicing, often impaired in malignancies [92,93]. Adamia et al. [94] proved that aberrant splicing was a common characteristic of AML and approximately 29% genes, encoding oncogenes, tumor suppressors, proteins implicated in splicing, apoptosis, cell cycle regulation, cell proliferation and differentiation, were aberrantly spliced in AML, as compared to normal CD34+ BM cells. Alternative splicing of *BCL2* genes may result in the formation of protein isoforms exhibiting pro- or anti-apoptotic activity [18,55]. Based on the example of *BCL2L1*, we observed not only high variability of this gene expression in AML patients, but also different proportions of the *BCL2L1* isoforms in AML and control samples. It was reported that the splicing events that control the expression of isoforms of the BCL2 family proteins were regulated by the Splicing Factor 3b Subunit 1 (SF3B1) [95]. The presence of mutations in the *SF3B1* gene was confirmed in 3.8% and 48.9% of patients from the AMLCG cohort and AML patients with normal karyotype, respectively [96,97,98]. We did not detect any significant mutation in this gene in the group of AML patients studied here, but only three patients had a normal karyotype and in the case of half of the patients, the karyotype was unknown. Instead, we detected mutations in other genes encoding splicing-related proteins, e.g., *RBMX*, *CWC27*, *HNRNPH2* or *HNRPNHU*, and correlated their presence with the expression of the selected BCL2 family members.

Regardless of the causes of the altered gene expression, the protein is the executive factor. Unfortunately, the BCL2 family protein-level data are limited and usually based on immunodetection in cells or tissues. According to the Human Protein Atlas (www.proteinatlas.org accessed 5 June 2021), only two family members (BID and BAX) were detected in blood by mass spectrometry. In BM samples, high protein level was noted only for BCL2 and BNIP2 and this does not correlate with the RNA levels (Table S4). The BM level of 13 proteins from BCL2 family was described in HPA as medium, 5 as low and six other proteins were not detected or have no data. In one of our earlier studies [99], we analyzed PB and BM proteomes of AML patients studied here, and no member of the BCL2 family was detected. Another problem is the BCL2 family proteins do not act alone, but are implicated in interactions with the family members and other partners, influenced also by their cellular localizations [100].

In the context of therapy, studies on the BCL2 family are of particular importance. In the light of numerous reports cited above, it seems that AML resistance to induction/consolidation therapy is a result of a complex network of protein interactions, in particular between the pro-survival and pro-death BCL2 family members. It has been shown that BCL-XL is about 10 times more efficient than BCL2 in preventing doxorubicin-induced apoptosis [101]. Inhibition of BCL-XL induced apoptosis and enhanced the effectiveness of chemotherapeutic agents in colorectal cancer cell lines [77]. Recently, a number of therapeutics targeting the BCL2 family has already been introduced into treatment or is now under investigation. One of the first evaluated drugs was ABT-737 (Venetoclax, BCL2-specific BH3 mimetic), a small molecule with high binding affinity to BCL-XL, BCL2, and BCL-W [102,103]. However, some inhibitors may elicit undesirable side-effects, as reported for the BCL-XL inhibition on platelets [60]. This is why future targeted therapies should be directed at specific targets and particular cell types, for example, leukemic cells and leukemic stem cells. In our opinion, a complex analysis of the impact of the BCL2 family proteins, also on transcript levels, on the AML pathogenesis should precede the introduction of a specific therapy.

Another aspect of the BCL2 family expression studies is connected with prognosis. Initial reports confirmed worse progression-free and overall survival of AML patients with high BCL2 expression in BM leukemic cells [104,105]. Del Poeta et al. [106] showed that a high BAX/BCL2 ratio level in the blast cells of de novo AML patients treated with standard induction and consolidation therapy was associated with a longer overall survival and disease-free survival. In addition, recently published data indicate that a high *BCL2* mRNA level after induction therapy or at complete hematologic remission negatively affects the AML outcome [88]. On the contrary, low *BCL2* level in the leukemic cells may help to identify patients with a favorable prognosis. It was shown that the absence of the *BCL2* expression characterized a subgroup of AML patients with distinct molecular and clinical characteristics, including low BM blast percentage, low *WT1* expression, underrepresentation of *FLT3* mutations, positive response to chemotherapy, and better OS [107]. However, we did not observe a correlation between the *BCL2* expression and OS. A similar conclusion was drawn by Zhou et al. [27] who noted an upregulation of *BCL2* in AML, but without any impact on the patient outcome. Bilbao-Sieyro et al. [88] documented a marginal association between a high *BCL2* level at the time of AML diagnosis and worse progression-free survival, but not with OS. The association with the negative outcome was stronger when a high *BCL2* level was detected directly after induction therapy or at CR. Our results indicate a higher impact of other genes of the *BCL2* family, in particular *BIK* and *BCL2L13*, on the AML patient outcome. Unexpectedly, *BCL2L1* was the only gene which presented inverse correlation (low level associated with worse prognosis). Similar observation was made by Andreeff et al. [108] who associated low *BCL2* content with poor survival in a group of patients with poor prognosis cytogenetics.

Summarizing, the following conclusion may be drawn from the literature data and results presented here: overexpression of anti-apoptotic *BCL2* family genes and underexpression of pro-apoptotic genes may facilitate and accelerate tumor development, and support tumor cell viability, but it is rather not the main driver of oncogenesis [109]. Although the gene expression results cannot be directly transposed to protein-level, they may be particularly useful in designing further research as well as diagnostic and therapeutic strategies.

## 5. Conclusions

In conclusion, the comprehensive analysis of gene expression data presented here revealed a complex dysregulation of the *BCL2* family in AML. Correlations with different factors, including other gene expressions, and the presence of mutations or patient outcome, was found for a number of the *BCL2* family members, both, pro- and anti-apoptotic. Definitely, factors other than gene expression and mutational status contribute to the BCL2 family function—posttranslational modifications, intracellular localization, stabilization, affinities, and interactions with other proteins. Despite this, gene expression studies undoubtedly contribute to the research on basic cellular processes in which the BCL2 family is engaged. Moreover, gene expression studies have a high potential of translation to clinical practice. First of all, they may support the selection of treatment targets. Secondly, post-treatment monitoring of the *BCL2* family gene expression should allow the estimation of treatment efficiency. In addition, high differences in the gene expression which we observed between individual patients suggested that not all of them might benefit from a BCL2-targeted therapy. In the end, the level of expression of particular genes from the *BCL2* family may predict the patient outcome at the time of AML diagnosis.

## Figures and Tables

**Table 1:** The summarized characteristics of AML patients included in the study.

Group	Number of Patients (F/M)	FAB Type (Number)	Age Median (Range)	WBC Count [×10^9^/L] Mean (Range)	*NPM1* mut.	*FLT3* mut.	*RUNX1/RUNX1T1*	Karyotype
CR	11 (6/5)	M1 (3) M2 (8)	52 (19–64)	36.3 (3.6–111)	2	2	2	normal (1) aberrant (4) NA (6)
RES	8 (3/5)	M1 (2) M2 (6)	53.5 (21–64)	62.17 (1.3–146)	2	1	1	normal (1) aberrant (4) NA (3)
X	8 (4/4)	M1 (4) M2 (4)	51 (25–65)	90.43 (19.9–233)	3	1	-	normal (1) aberrant (2) NA (5)
All patients	27 (13/14)	M1 (9) M2 (18)	52 (19–65)	60.0 (1.3–233)	7	4	3	normal (3) aberrant (10) NA (14)

AML patients were divided into three groups according to their response to the treatment (CR—complete remission; RES—resistance to therapy; X—death during therapy). *FLT3* mut. = *FLT3*-ITD (internal tandem duplication in the *FLT3* gene); *RUNX1/RUNX1T*—fusion gene, result of t(8;21) translocation; NA—data not available.

**Table 2:** Comparison of gene expression ratio for two pairs of genes: *BCL2A1* and *BCL2L1*; *BCL2* and *BCL2L11* in AML and control samples.

Group	*BCL2A1/BCL2L1* Ratio	*BCL2/BCL2L11* Ratio
AML—mean ratio (st. dev.) CR—mean ratio (st. dev.) RES—mean ratio (st. dev.)	1.15 (1.02)	3.26 (2.53)
	1.31 (0.7)	2.93 (2.43)
	0.92 (1.44)	3.16 (3.05)
X—mean ratio (st. dev.) ctrl—mean ratio (st. dev.)	1.15 (0.99)	3.81 (2.3)
	5.67 (4.56)	0.83 (0.50)
AML vs. ctrl * CR vs. ctrl*	**0.0034**	**0.033**
	**0.0110**	0.1264
RES vs. ctrl * X vs. ctrl *	**0.0485**	0.1333
	**0.0121**	**0.0121**
RES vs. X *	0.5054	0.3823
RES vs. CR *	**0.0506**	0.9678
CR vs. X *	0.5448	0.3950

* *p* value (Wilcoxon rank sum exact test; statistically significant values are in bold); st. dev.—standard deviation.

**Table 3:** Mean values of the expression levels of the *BCL2L1* gene isoforms, extracted from the RNA-seq data.

*BCL2L1* Gene Isoform	Length of the Gene (Protein)	CR—Mean TPM (st. dev.)	RES—Mean TPM (st. dev.)	X—Mean TPM (st. dev.)	Ctrl—Mean TPM (st. dev.)
**ENST00000307677**	**2574 bp (233 aa)**	**39.14 (32.37)**	**101.71 (140.27)**	**48.01 (56.36)**	**14.90 (4.68)**
**ENST00000376055**	**2496 bp (170 aa)**	**7.05 (8.13)**	**26.29 (50.13)**	**6.89 (7.52)**	**6.32 (5.18)**
**ENST00000376062**	**2578 bp (233 aa)**	**4.49 (3.26)**	**5.55 (5.80)**	**4.67 (4.93)**	**3.39 (2.95)**
ENST00000420488	2444 bp (233 aa)	0.41 (0.45)	0.75 (0.88)	0.22 (0.26)	0.11 (0.19)
**ENST00000422920**	**2227 bp (170 aa)**	**1.82 (0.91)**	**5.89 (8.84)**	**2.44 (2.62)**	**0.97 (0.15)**
ENST00000434194	2441 bp (233 aa)	0.10 (0.25)	0.11 (0.19)	0.23 (0.45)	0.16 (0.28)
ENST00000439267	2426 bp (233 aa)	0.48 (0.53)	0.48 (0.51)	0.63 (0.67)	0.25 (0.22)
ENST00000450273	1292 bp (307 aa)	0.35 (0.50)	0.51 (0.52)	0.48 (0.59)	0.76 (0.40)
ENST00000456404	2562 bp (233 aa)	0.97 (0.63)	0.72 (0.83)	1.20 (0.95)	0.87 (0.47)

Legend: CR—complete remission; RES—resistance to therapy; X—death during therapy; SD—standard deviation; TPM—transcripts per million; st. dev.—standard deviation. Dominant gene isoforms presented in Figure 3 are in bold.

**Table 4:** The associations between the expression levels of the *BCL2* family genes and other factors, detected by MANOVA, in the studied AML patients. * *p* < 0.05, ** *p* < 0.01, *** *p* < 0.001.

Expression of Genes	Associated Factor	*p* Value
*BCL2L1*, *BID*, *BOK*, *HRK*	*BRCA2*_status, *RUNX1*_status	4.145 × 10^−10^ *** 0.04287 *
*BCL2L1*, *BID*, *BOK*, *HRK*	*BRCA2* status	2.106 × 10^−9^ ***
*BCL2L1*, *BID*, *HRK*	*RUNX1* status	0.0002187 ***
*BCL2L10*, *BCL2L2*, *HRK*	*HNRNPU* status	8.038 × 10^−7^ ***
*BCL2L1*, *BBC3*, *BOK*, *BCL2L10*, *BCL2L11*	*HNRNPH2* status	1.129 × 10^−5^ ***
*BCL2L11*, *BCL2L13*, *BID*, *BIK*, *BNIP1*	WBC count	6.459 × 10^−5^ ***
*HRK*, *BAK1*, *BCL2A1*, *BID*, *BNIP1*	*SNRNP200* status	0.002168 **
*BCL2L10*, *BNIP2*	*DDX42* status	0.000545 ***
*BCL2*, *BCL2A1*, *BCL2L10*, *BID*, *BNIP1*, *MCL1*	*CWC27* status	0.007744 **
*BMF*, *RTL10*, *BAX*, *BCL2L11*, *BCL2L2*	*CEBPA* status	0.00383 **
*BCL2L13*, *BIK*, *PMAIP1*	*RBMX* status	0.0004931 ***
*MCL1*, *BCL2L11*	*HNRNPUL1* status	0.05703
*BCL2L2.PABPN1*, *BNIP2*	*DHX38* status	0.009772 **
*BCL2*, *BCL2L11*, *BCL2L12*, *BCL2L15*	*EP300* status	0.04793 *
*BCL2L13*, *BCL2L2*, *BIK*	*ATM* status	0.003997 **
*BAX*, *BCL2A1*, *BNIP2*	*KRAS* status	0.01254 *
*BCL2L13*, *BCL2L2*	*DNMT3A* status	0.02401 *
*BNIP1*, *BCL2L2.PABPN1*	*IDH1* status	0.01978 *
*BBC3*, *BCL2L1*, *BCL2L11*, *BOK*, *MCL1*	*IDH2* status	0.02641 *
*BCL2L1*, *BOK*	*IDH2* status, *MCL1* status	0.001286 ** 0.007325 **
*BCL2L1*, *BOK*	*IDH2* status, *MCL1* status, *BRCA2* status	1.755 × 10^−5^ *** 4.508 × 10^−5^ *** 4.624 × 10^−6^ ***
*BCL2L1*, *BOK*	*MCL1* status	0.001242 **
*BAD*, *BAK1*, *BCL2L11*, *BID*, *BNIP2*, *BNIP3*	*RUNX1-RUNX1T1* status	0.002101 **
*BCL2L13*, *BID*, *BIK*, *BNIP1*	*NPM1* status	0.02555 *
*BAD*, *BAX*, *BCL2L1*, *BCL2L11*, *BCL2L13*, *BCL2L2.PABPN1*, *BID*, *BIK*, *BNIP1*, *BOK*	Blast percentage in PB	0.003099 **
*BCL2L1*, *BCL2L11*, *BCL2L15*, *BCL2L2.PABPN1*, *BID*, *BIK*, *BNIP1*, *BOK*, *HRK*	Blast percentage in BM	0.000261 ***
*BAK1*, *BBC3*, *BCL2L1*, *BCL2L11*, *BCL2L12*, *BOK*	Tissue type (PB, BM)	0.0004833 ***
*BCL2L15*, *BCL2L2*, *BNIP1*, *BNIP3*	FAB type (M1, M2)	0.03326 *

## Data Availability

All data generated or analyzed during this study are included in this published article and its supplementary information files.

## Supplementary Materials

The following are available online at https://www.mdpi.com/article/10.3390/cancers13133175/s1, Figure S1: Expression levels of 26 genes from the *BCL2* family in AML (27 patient samples from this study (TS)) versus control samples (ctrl) which included controls from this study and the additional controls from Human Protein Atlas (HPA), Figure S2: Graphical presentation of Spearman correlation of gene expression calculated for each pair of the genes encoding *BCL2* family proteins, based on RNA-seq data collected for control samples (A,C) and AML patients as a whole group (B,D), Figure S3: Graphical presentation of Spearman correlation of gene expression calculated for each pair of the genes encoding BCL2 family proteins, based on RNA-seq data collected for AML divided into three subgroups according to the response to therapy: CR (A,D), RES (B,E) and X (C,F), Figure S4: Overall survival (OS, A–E) and disease free survival (DFS, F-L) of 27 AML patients divided according to the WBC count (F) or the expression level of the following genes: *BCL2L2* (A,H); *BCL2L12* (B,I); *BAX* (C,J); *BID* (D,K); *BCL2L1* (E,L); *WT1* (G), Figure S5: The mutation pattern in genes encoding proteins from mRNA Splicing-Major Pathway, Figure S6: The graphical presentation of *BCL2* family genes whose expression was associated with *RUNX1* mutation status by MANOVA, Figure S7: The graphical presentation of *BCL2* family genes whose expression was associated with *BRCA2* mutation status by MANOVA, Figure S8: The levels of expression of *BCL2A1* gene, *NFKB1* and *NFKBIA* genes encoding two transcription factors which are described to regulate *BCL2A1* expression, and *BID* gene, encoding BH3-only partner of BCL2A1 protein, Table S1: The list of samples included into analysis, Table S2: Results of differential expression analysis of genes from *BCL2* family (yellow background) and genes encoding proteins interacting with BCL2 family (blue background), Table S3: Expression levels (in TPM) of genes from BCL2 family (yellow background) and genes encoding proteins interacting with BCL2 family (blue background) in particular samples, Table S4: Data extracted from the Human Protein Atlas, Table S5: Isoforms of *BCL2L1* gene, Table S6: Correlation of expression levels (in TPM) of *BCL2L1* gene and the genes encoding proteins interacting with BLC2L1, indicated by STRING (https://string-db.org, accessed on 2 February 2019), in AML samples resistant to therapy (RES), Table S7: Mutations identified in *BCL2* family genes and the genes encoding proteins interacting with BCL2L1, annotated with the use of eVai software (www.engenome.com/evai/, accessed on 2 February 2019), Table S8: Mutations identified in *BCL2* family genes and the genes encoding proteins interacting with BCL2L1, annotated with the use of eVai software (www.engenome.com/evai/, accessed on 2 February 2019), Table S9: Results of uni- and multivariate analysis of relations between the levels of *BCL2* family gene expression and other features.
