# Supplementary material for: Transcript-Level Dysregulation of BCL2 Family Genes in Acute Myeloblastic Leukemia
Source: Cancers (Basel). 2021 Jun 25;13(13):3175. doi: 10.3390/cancers13133175 (PMC8267690; doi:10.3390/cancers13133175)
Supplement: Supplementary file 1 [file cancers-13-03175-s001.zip › cancers-1234055-supplementary/cancers-1234055-supplementary.pdf]

cancers MDPI

Transcript-Level Dysregulation of BCL2 Family Genes in Acute Myeloblastic Leukemia

Luiza Handschuh, Pawel Wojciechowski, Maciej Kazmierczak and Krzysztof Lewandowski

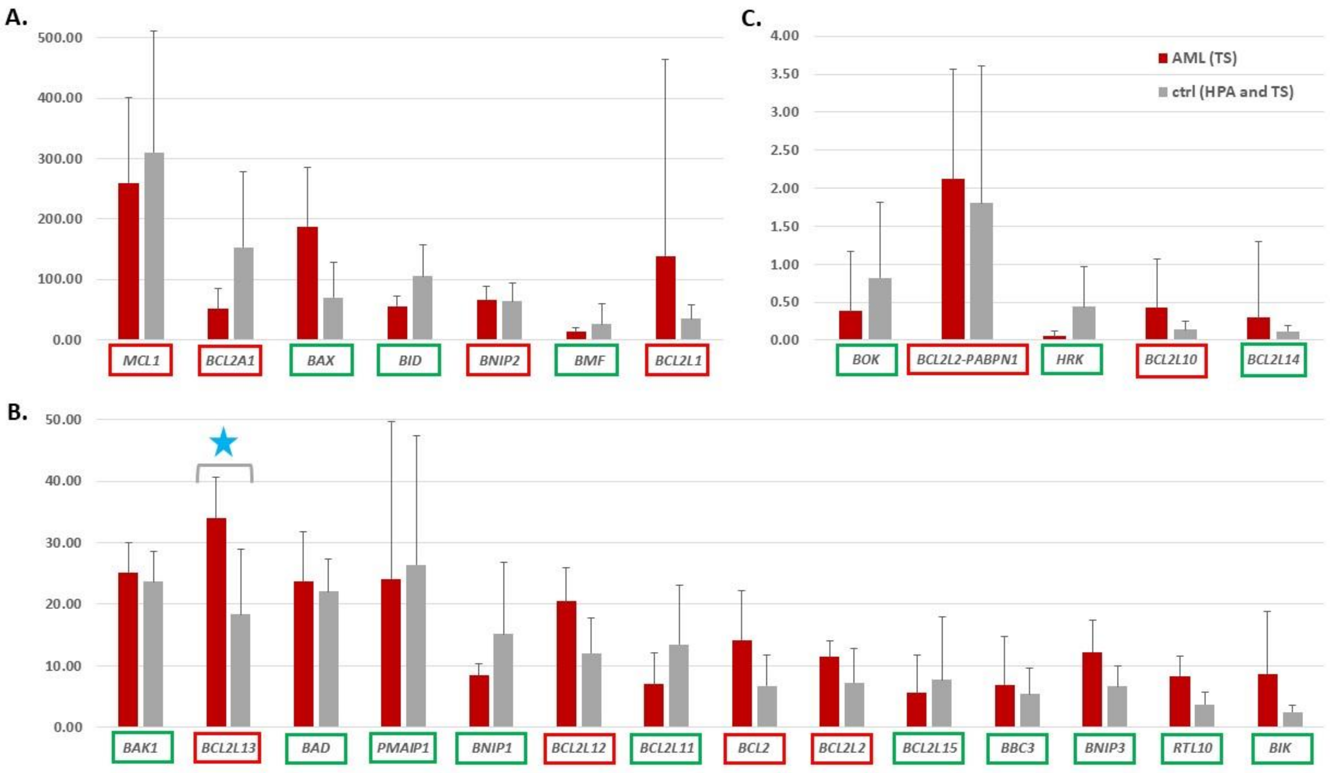

Figure S1. Expression levels of 26 genes from the *BCL2* family in AML (27 patient samples from this study (TS)) versus control samples (ctrl) which included controls from this study and the additional controls from the Human Protein Atlas (HPA). The expression levels come from the RNA-seq data and are presented as mean TPM (transcripts per million) values with standard deviation bars (*y* axis). For clarity, the genes are presented at the same order as in the Figure 1 and divided into three separate graphs, from (**A**) the highest to (**B**) the middle and (**C**) the lowest gene expression. The genes encoding the pro-apoptotic proteins are indicated in green, the anti-apoptotic are in red frames. Blue star indicate the only one gene with statistically significant expression change between AML and ctrl (Bonferroni-corrected p value < 0.0019). To compare the expression levels Mann-Whitney test was applied.

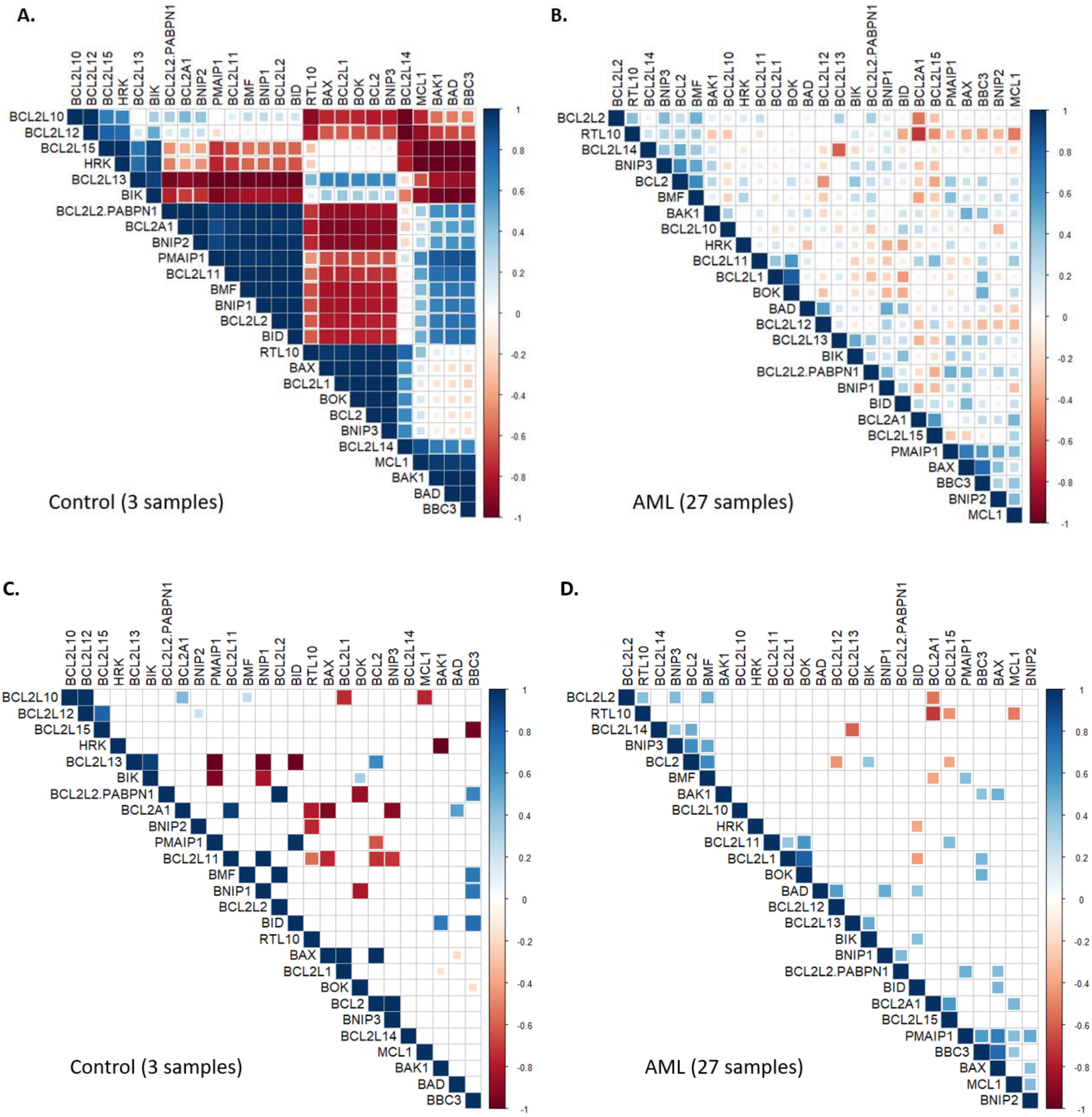

Figure S2. Graphical presentation of Spearman correlation of gene expression calculated for each pair of the genes encoding BCL2 family proteins, based on RNA-seq data collected for control samples (**A,C**) and AML patients as a whole group (**B,D**). Color intensity and the size of the squares are proportional to the correlation coefficients. Panels (**A,B**) present all correlations whereas panels (**C,D**) only statistically significant correlations.

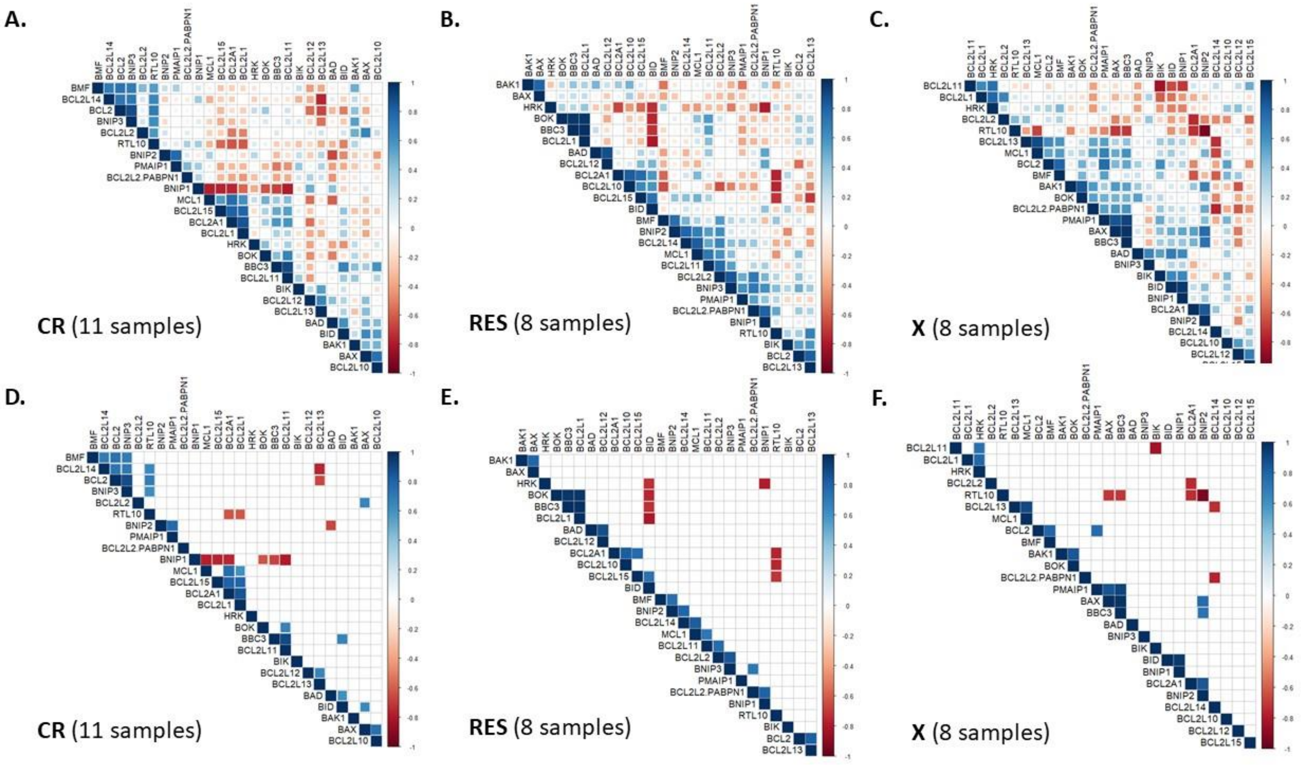

Figure S3. Graphical presentation of Spearman correlation of gene expression calculated for each pair of the genes encoding BCL2 family proteins, based on RNA-seq data collected for AML divided into three subgroups according to the response to therapy: CR (**A,D**), RES (**B,E**) and X (**C,F**). Color intensity and the size of the squares are proportional to the correlation coefficients. Panels (**A**–**C**) present all correlations whereas panels (**D**–**F**) only statistically significant correlations.

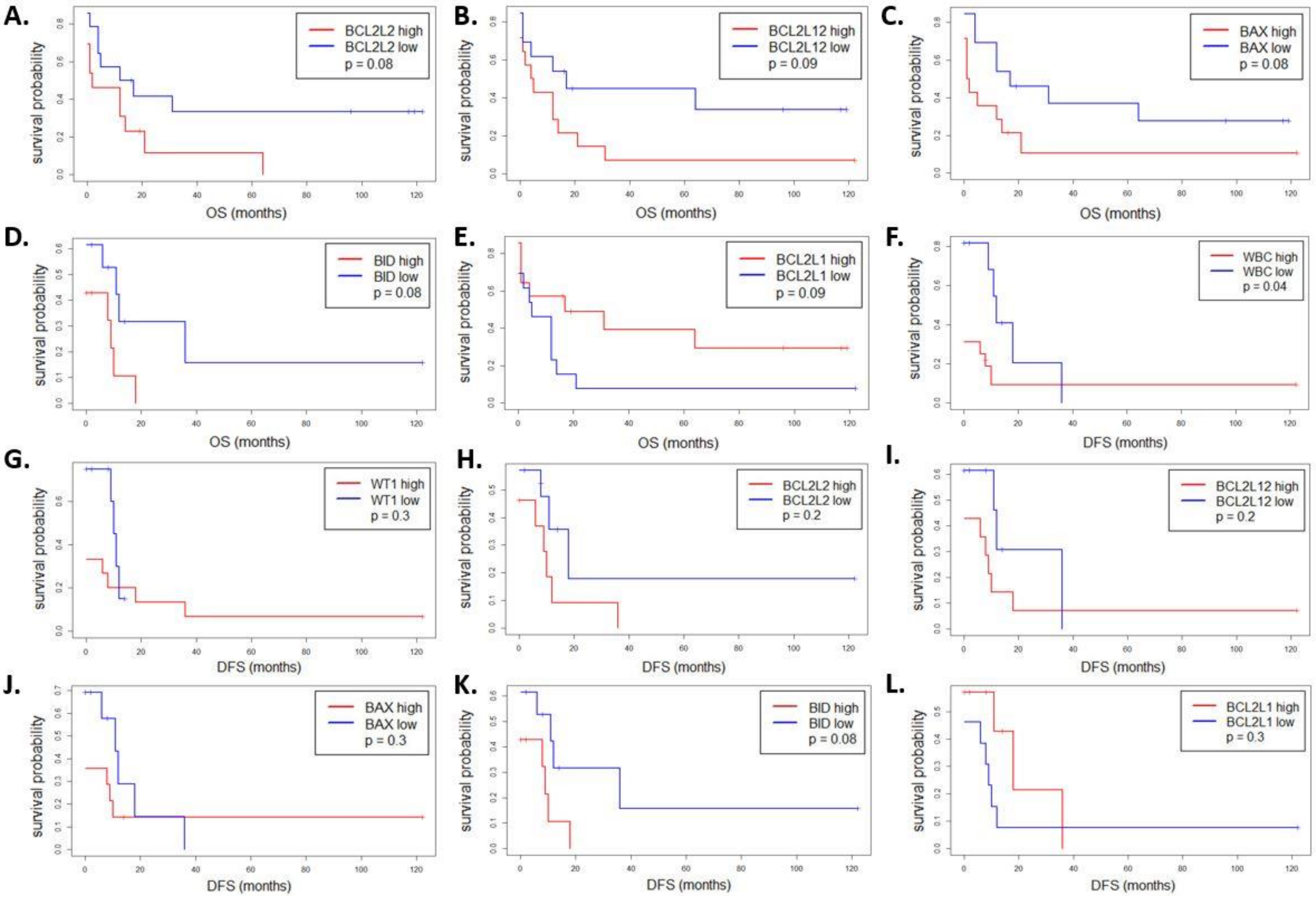

Figure S4. Overall survival (OS, **A**–**E**) and disease free survival (DFS, F-L) of 27 AML patients divided according to the WBC count (**F**) or the expression level of the following genes: *BCL2L2* (**A**,**H**); *BCL2L12* (**B**,**I**); *BAX* (**C**,**J**); *BID* (**D**,**K**); *BCL2L1* (**E**,**L**); *WT1* (**G**). In each case, blue curve means low level and red curve high. Log-rank test p values are shown (< 0.05 indicate statistically significant differences between two curves).

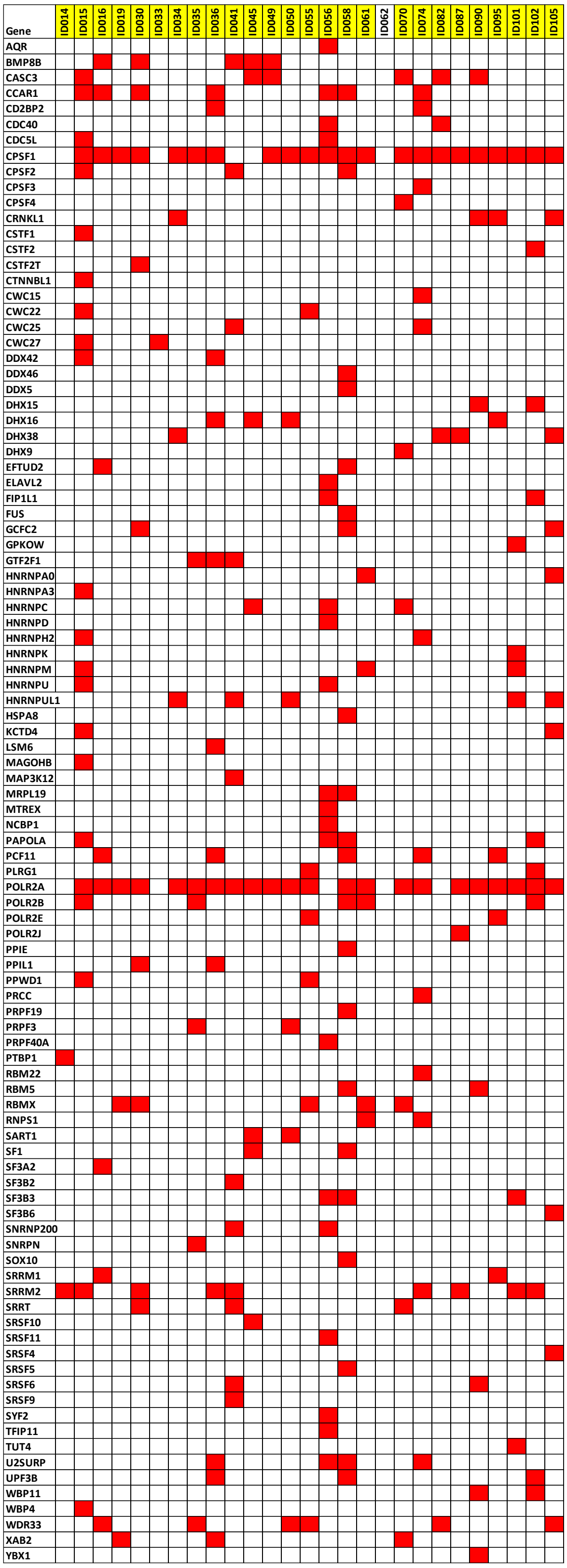

Figure S5. The mutation pattern in genes encoding proteins from mRNA Splicing-Major Pathway (downloaded from https://pathcards.genecards.org/Pathway/2004 accessed on 24 March 2021). 98 genes with mutation in a least one sample are shown, ranked in an alphabetic order. The presence of mutation is indicated by a red square. Supplementary Table 7 contains the full list of variants detected in the analyzed genes.

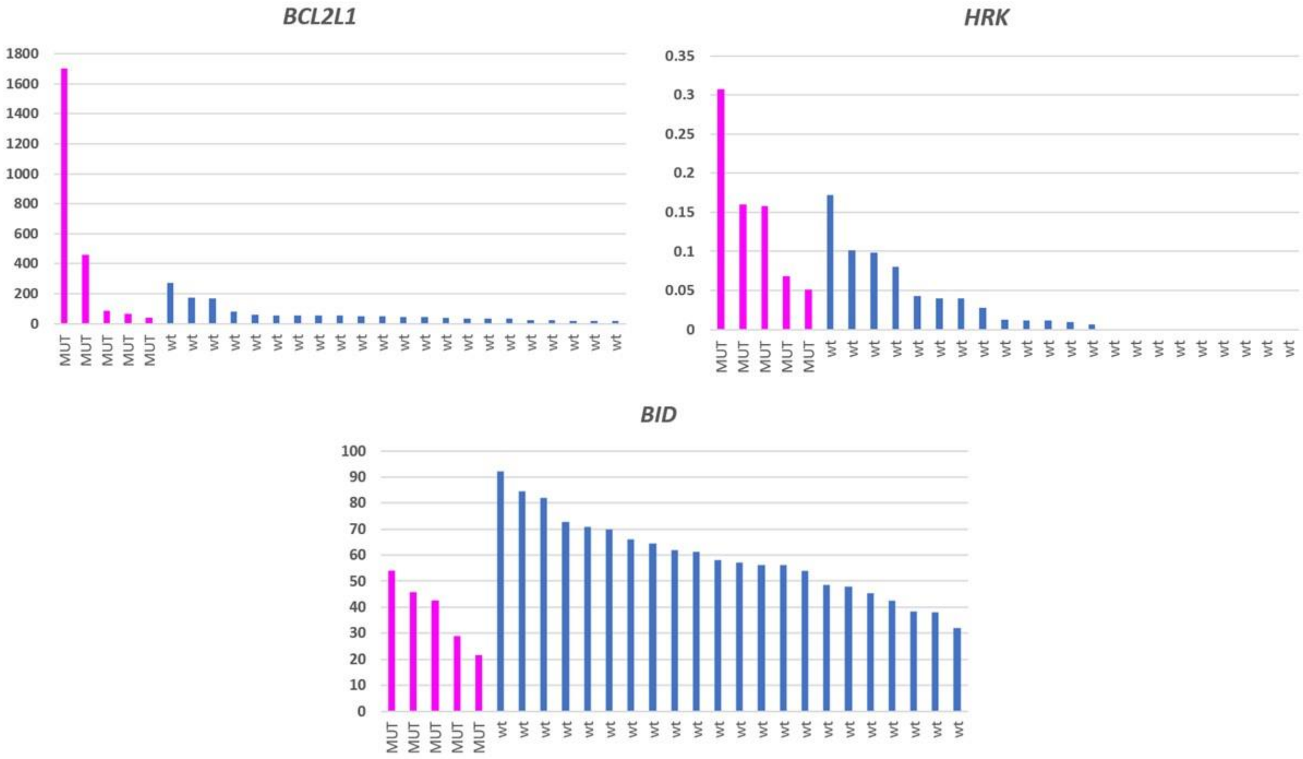

BCL2L1, BID, HRK	RUNX1_status	0.0002187 ***

Figure S6. The graphical presentation of *BCL2* family genes whose expression was associated with *RUNX1* mutation status by MANOVA. Expression level, extracted from RNA-seq data, is presented in TPMs (transcripts per million) on the *Y*-axis. Samples on the *X*-axis are divided into 2 subgroups, with the presence of the mutation (MUT) or wild-type (wt). *** $p < 0.001$.

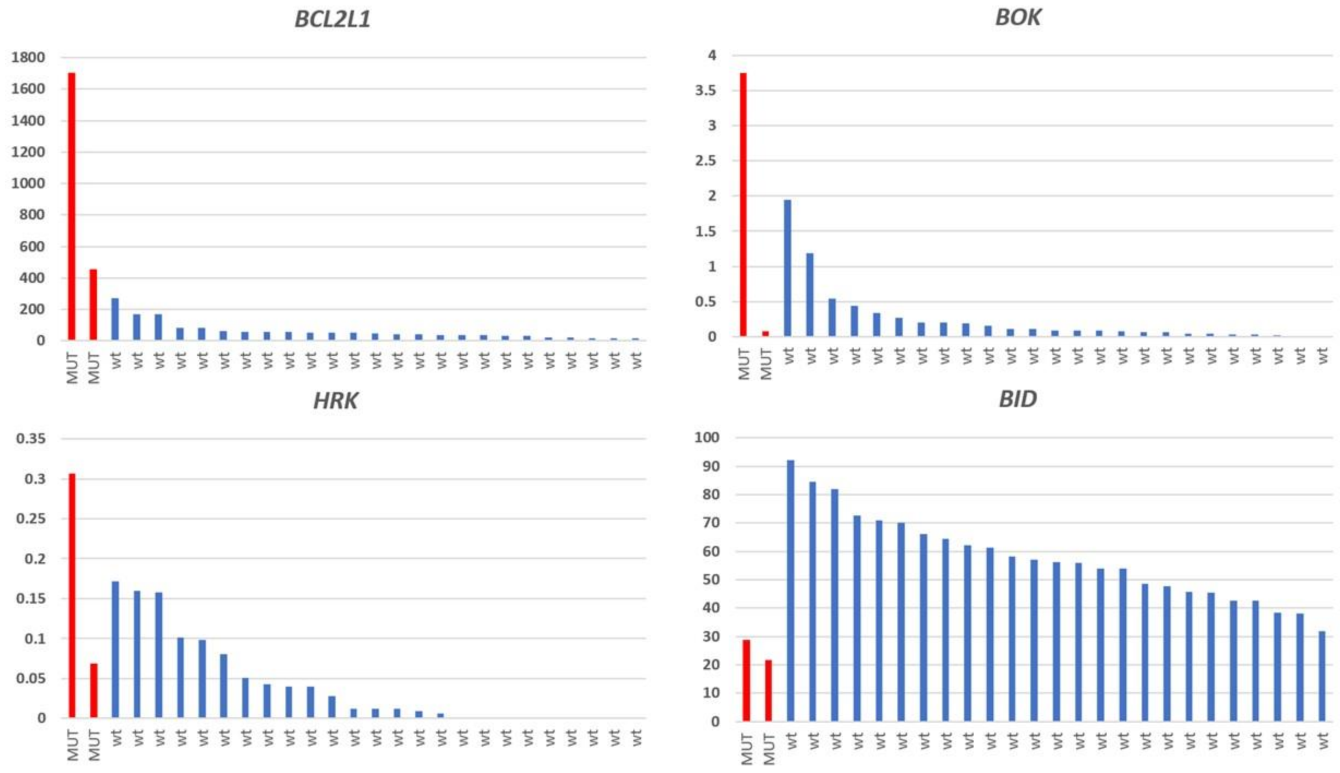

BCL2L1, BID, BOK, HRK	*BRCA2* _status	2.106×10^{-9} ***

Figure S7. The graphical presentation of *BCL2* family genes whose expression was associated with *BRCA2* mutation status by MANOVA. Expression level, extracted from RNA-seq data, is presented in TPMs (transcripts per million) on the *Y*-axis. Samples on the *X*-axis are divided into 2 subgroups, with the presence of the mutation (MUT) or wild-type (wt). *** $p < 0.001$.

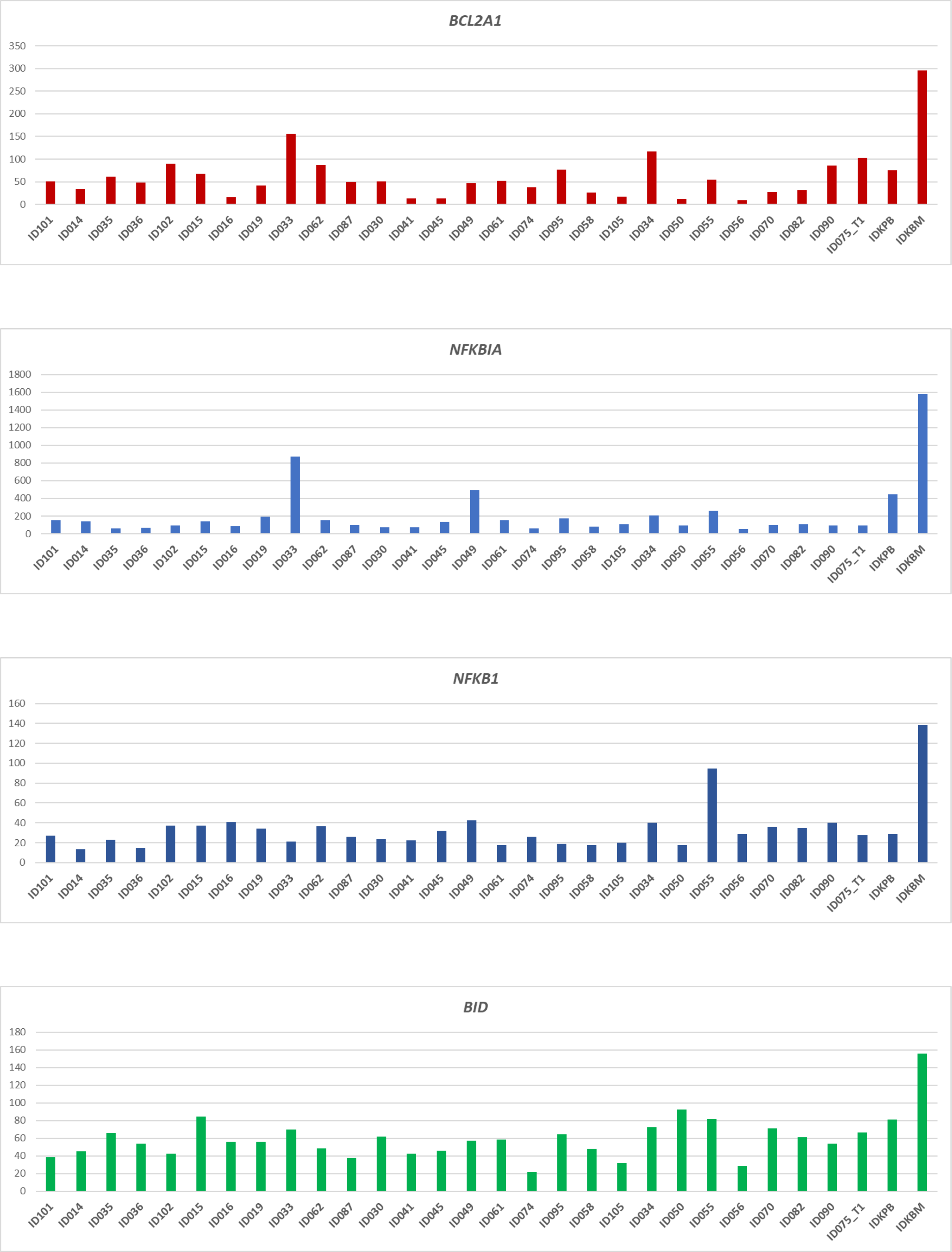

Figure S8. The levels of expression of *BCL2A1* gene, *NFKB1* and *NFKBIA* genes encoding two transcription factors which are described to regulate *BCL2A1* expression, and *BID* gene, encoding BH3-only partner of BCL2A1 protein. Expression levels, extracted from RNA-seq data, are presented in TPMs (transcripts per million) on the Y-axis. Samples are presented on the X-axis. Three samples from the right are control samples. In the case of all four genes, the highest level was detected for healthy BM sample, presented as a first from the right. The Pearson correlation coefficients calculated for TPMs of particular gene pairs are as follows: 0.85 for *BCL2A1* and *NFKBIA*; 0.67 for *BCL2A1* and *NFKB1*; 0.71 for *BCL2A1* and *BID*; 0.73 for *BID* and *NFKBIA*; 0.70 for *BID* and *NFKB1*; 0.71 for *NFKBIA* and *NFKB1*.
